# Effectiveness of family metacognitive training in mothers with psychosis and their adolescent children: a multicenter study protocol

**DOI:** 10.3389/fpsyg.2024.1359693

**Published:** 2024-03-22

**Authors:** Susana Ochoa, Victoria Espinosa, Raquel López-Carrilero, Irene Martinez, Alejandro De Haro Barrera, Irene Birulés, Ana Barajas, Trinidad Pélaez, Luciana Díaz-Cutraro, Marta Coromina, Alexandre González-Rodríguez, Marina Verdaguer-Rodríguez, Alfonso Gutiérrez-Zotes, Carolina Palma-Sevillano, Cristian Montes, Judith Gallego, Beatriz Paya, Francesc Casanovas, María Roldán, Emma Noval, Paloma Varela Casals, Miriam Salas-Sender, Ana Aznar, Rosa Ayesa-Arriola, Esther Pousa, Manuel Canal-Rivero, Nathalia Garrido-Torres, Clara Montserrat, Laura Muñoz-Lorenzo, Josep María Crosas

**Affiliations:** ^1^Parc Sanitari Sant Joan de Déu, Sant Boi de Llobregat, Spain; ^2^Centro de Investigación Biomédica en Red de Salud Mental, Instituto de Salud Carlos III, Madrid, Spain; ^3^Fundació Sant Joan de Déu, Esplugues de Llobregat, Barcelona, Spain; ^4^Etiopatogènia i tractament dels trastorns mentals greus (MERITT), Institut de Recerca Sant Joan de Déu, Esplugues de Llobregat, Barcelona, Spain; ^5^Facultat de Psicologia Departament de Cognició, Desenvolupament i Psicologia de l'Educació, Universistat de Barcelona, Barcelona, Spain; ^6^Departament de Psicologia, Facultat de Psicologia Clínica I de la Salut. Serra Húnter Programme, Universitat Autònoma de Barcelona, Barcelona, Spain; ^7^Department of Psychology, FPCEE Blanquerna, Universitat Ramon Llull, Barcelona, Spain; ^8^Department of Mental Health, CIBERSAM, Mutua Terrassa University Hospital, Fundació Docència i Recerca Mutua Terrassa, University of Barcelona, Terrassa, Spain; ^9^Department of Clinical and Health Psychology, Universitat Autònoma de Barcelona, Cerdanyola del Vallès, Barcelona, Spain; ^10^Hospital Universitari Institut Pere Mata, Institut d’Investigació Sanitària Pere Virgili-CERCA, Universitat Rovira i Virgili, Reus, Spain; ^11^Hospital de Mataró, Consorci Sanitari del Maresme, Barcelona, Spain; ^12^Virgen del Rocío University Hospital, Network Centre for Biomedical Research in Mental Health (CIBERSAM), Institute of Biomedicine of Seville (IBiS), First-episode Psychosis Research Network of Andalusia (Red PEPSur), University of Seville, Seville, Spain; ^13^Fundació Vidal i Barraquer, Barcelona, Spain; ^14^Department of Psychiatry, Marqués de Valdecilla University Hospital, IDIVAL. School of Medicine, University of Cantabria, Santander, Spain; ^15^Hospital del Mar Medical Research Institute (IMIM) of Barcelona, Autonomous University of Barcelona, Barcelona, Spain; ^16^Centre d’Higiene Mental Les Corts, Barcelona, Spain; ^17^Department of Psychiatry, Hospital de la Santa Creu i Sant Pau, Institut d’Investigació Biomèdica-Sant Pau (IIB-Sant Pau), Barcelona, Spain; ^18^Departamento de Psiquiatría, IIS-Fundación Jiménez Díaz, Madrid, Spain; ^19^Department of Mental Health, Hospital Universitari Parc Taulí, Institut d’Investigació i Innovació Parc Taulí I3PT, Universitat Autònoma de Barcelona, Sabadell, Catalonia, Spain

**Keywords:** mothers with psychosis, adolescents’ mental health, metacognitive training, family intervention, protocol, clinical trial

## Abstract

**Background:**

More than half of women with psychosis take care of their children despite the difficulties caused by the disease. Additionally, these kids have a higher risk of developing a mental health disorder. However, no interventions have been developed to meet these needs. Metacognitive Training (MCT) is a psychological intervention that has demonstrated its efficacy in improving cognitive insight, symptom management and social cognition in people with first-episode psychosis (FEP). Additionally, MCT has shown better results in women than men with FEP. This study aims to adapt and evaluate the efficacy of MCT-F in mothers and adolescent children in an online group context with the main purpose of improving family relationships, cognitive awareness and symptoms in women with psychosis and increase their children’s knowledge of the disease and their functioning. As secondary objectives, it also aims to evaluate improvements in metacognition, social cognition, symptoms, protective factors and self-perception of stigma.

**Materials and methods:**

A quasi-experimental design with participants acting as their own control will be carried out. Forty-eight mothers with psychosis and their adolescent children (between 12 and 20 years old) recruited from a total of 11 adult mental health care centers will receive MCT-F. Participants will be evaluated 11 weeks before the intervention (T1), at baseline (T2), and post-intervention (T3) with a cognitive insight scale, as a primary outcome. Measures of metacognitive and social cognition, symptoms, cognitive functioning, family and social functioning, protective factors (self-esteem, resilience, and coping strategies) and self-perceived stigma will be addressed as secondary outcomes. Assessment will also address trauma and attachment in mothers and, lastly, the feasibility and acceptability of MCT-F in both participant groups.

**Discussion:**

This will be the first investigation of the efficacy, acceptability, and viability of the implementation of MCT-F. The results of this study may have clinical implications, contributing to improving mothers’ with psychosis and adolescents’ functioning and better understanding of the disease, in addition to the possible protective and preventive effect in adolescents, who are known to be at higher risk of developing severe mental disorders.

**Clinical trial registration:**https://clinicaltrials.gov/, identifier [NCT05358457].

## 1 Introduction

### 1.1 Women and motherhood in schizophrenia and other psychosis

Schizophrenia is a chronic disease with a heterogeneous course. Several factors have been described as influencing the course of the illness, among them, gender has been found to be a determinant factor (Ochoa et al., 2006). Previous research suggests that women with psychosis have fewer admissions to acute psychiatric units and higher frequencies of diagnoses of schizoaffective or delusional disorders than males (Iniesta et al., 2012) as well as a better premorbid adjustment, course of the illness, social functioning, and treatment response than males (Ochoa et al., 2012; Riecher-Rössler et al., 2018; Seeman, 2020). In this line, more than 60% of women with psychosis are married (Usall, 2001), and between 50 and 60% are mothers (McGrath et al., 1999; Howard et al., 2001), which is a similar percentage to that found in women without a mental disorder diagnosis (Haukka et al., 2003).

Although motherhood has been explored in other diagnoses, little has been studied in mothers with schizophrenia and most of them are centered in pregnancy and post-partum, leading to the development of specialized mother-baby units (Wan et al., 2008; Gentile and Fusco, 2019). In previous research, it was found that mothers with schizophrenia express significantly more need in taking care of their children than men (Ochoa et al., 2001). Indeed, a specific instrument was developed for the assessment of their specific needs (CAN-M) (Howard et al., 2007). The difficulties of mothers with schizophrenia could be related to several aspects such as illness severity, problems in reasoning biases, attributional errors and social cognitive impairments, presence of social stressors, self-stigma associated with the illness, and a lack of protective factors to deal with them (Wan et al., 2008). In fact, custody loss is a main fear for many of these mothers (Dolman et al., 2013) and is experienced more often than in parents without mental health problems (Kaplan et al., 2019).

### 1.2 Adolescent children of mothers with schizophrenia

Adolescence is one of the most difficult and crucial periods in life, when the importance of relating to others and self-reflection is highly present (Weil et al., 2013; Arnett, 2023). Moreover, during adolescence, social cognition-emotional recognition, theory of mind, attributional style, and social perception become instrumental in social relationships and in the development of the social brain (Kilford et al., 2016; Steenhuis et al., 2020). On the other hand, adolescents whose mothers are diagnosed with psychosis have specific challenges. For instance, when a mother has developed psychosis, it can lead to difficulty expressing feelings to their children and the disorder can be treated as a family secret (Cooklin, 2013). However, it is well demonstrated that all children and adolescents need support to understand the disorder in order to avoid developing wrong beliefs and attributions to their mother’s potentially irrational behavior (Riebschleger et al., 2009). With this purpose, Blake et al. (2015) developed a guide for mothers with psychosis and their families with recommendations on how to talk about the illness and avoid experiencing negative states such as anxiety, anger, denial, sadness, and guilt.

Nevertheless, some women with psychosis have difficulty expressing feelings with their children that could derive from those unpredictable life situations and the family setting could become chaotic and confusing (Cudjoe et al., 2023). Aldridge and Becker (2003) found adolescents of mothers with psychosis often assume adult responsibilities. Moreover, they could identify with their mother and became hyper alert to their own behavior due to fears of developing the same symptoms (Cooklin, 2013). Thus, symptoms observed in these children can range from emotional and behavioral problems to cognitive impairments and social difficulties (Radley et al., 2022b), which become more evident in adolescence. Consequently, several studies have emphasized the importance of psychoeducation and coping skills to avoid isolation and promote resilience (Pitman and Matthey, 2004; Riebschleger et al., 2009).

### 1.3 Psychological interventions for mothers with psychosis and their adolescent children

Around 70% of children living with parents with mental health problems will experience mental health difficulties (Cooklin, 2010). Particularly, the estimated risk of developing schizophrenia is of approximately 10% in individuals who have a parent with psychosis, increasing to 50% if both parents are affected (Hannon et al., 2016). Moreover, Goldstein et al. (2013) reported that the heritability of psychosis is linked to the X chromosome, finding a higher prevalence of maternal history of the mental disorder. In the same line, Barajas et al. (2019) also found higher prevalence of delusions in FEP patients whose mothers have psychosis. This association may be due to genetic aspects, but also to environmental factors such as problems in family dynamics (Cudjoe et al., 2023).

Even though there are several programs addressed to high-risk adolescents, aimed to avoid the transition to psychosis (Schmidt et al., 2015), these are indicated when the adolescent has already presented subclinical symptoms or a decline in functioning. Given the evidence demonstrating the issues that adolescent children of mothers with psychosis face, they should receive interventions before the symptoms or decline in functioning are evident.

Conversely, limited research exists on developing and evaluating interventions specifically tailored to mothers with psychosis and their adolescent children (Wan et al., 2008). Recently, a systematic review (Tapias et al., 2021) aimed to explore psychological interventions addressed to children and adolescents whose parents have a mental disorder suggested that little research has been conducted in this topic. Although some studies included samples of people with psychosis, none of them addressed psychosis interventions and some studies’ samples included less than 5% of people with psychotic disorders. Of those studies, few studies specifically targeted mothers (Rosenblum et al., 2017) or the adolescent period (Pitman and Matthey, 2004; Fraser and Pakenham, 2008). Furthermore, although some interventions include parents and offspring together, only one intervention, kidstime project by Wolpert et al. (2015), included parents with psychosis in their sample. Although only 5 families were included in the study, the results show an improvement in their knowledge of the illness and better relationships in the family. However, interventions focusing solely on psychoeducation about parental mental illness or parenting skills have been shown to be insufficient in improving cognitive biases and social cognition (Wolpert et al., 2015; Marston et al., 2016),

Thus, the evidence of interventions is scarce, and we should highlight two clear deficiencies: (1) the lack of studies focused on mothers with psychosis and (2) the interventions available for mental disorders address increasing knowledge of the illness but none aim to improve errors in cognitive biases, social cognition or self-reflectiveness.

### 1.4 Metacognitive training for psychosis

Metacognitive training for psychosis (MCT) (Moritz and Woodward, 2007) is a low-threshold approach based on a cognitive behavioral intervention combined with psychoeducation. The intervention is based on a normalizing approach in which those cognitive biases more frequent in people with psychosis are commented on in an illustrative and entertaining way. MCT is freely available in 37 languages^1^ and other versions of the program have been developed for other disorders (depression, borderline personality and obsessive-compulsive disorders, among others). Studies suggests MCT might be more effective in enhancing cognitive insight compared to cognitive remediation (Balzan et al., 2019) treatment and usual (TAU) (Lam et al., 2015) or psychoeducational interventions (Ochoa et al., 2017; Ahuir et al., 2018). A 3-year follow-up randomized controlled trial involving a 3-year follow-up randomized controlled trial, found that MCT was more effective in improving quality of life and self-esteem compared to an active control group that received cognitive remediation (Moritz et al., 2014). A recent meta-analyze has shown that MCT is effective in reducing positive and negative symptoms and enhance cognitive biases, self-esteem, and functioning (Penney et al., 2022). Additionally, MCT has good acceptability and tolerability (Moritz et al., 2011). The Spanish Metacognition Group has also demonstrated the effectiveness of MCT in people with FEP, finding improvements in cognitive insight, symptoms, social cognition, and irrational beliefs (Ochoa et al., 2017; Birulés et al., 2020; Salas-Sender et al., 2020). Interestingly, it was found that women with psychosis benefited more from the MCT intervention than their male counterparts regarding general symptoms, cognitive insight and social cognition, suggesting that MCT is a gender sensitive intervention (Salas-Sender et al., 2020). Jointly addressing the metacognition of two family members could increase the effectiveness of the intervention, as it allows for the collaborative identification of cognitive biases as well as alternative interpretations and the shared use of intervention tools.

As a result of the COVID-19 pandemic, research has demonstrated the viability of the use of telemedicine to treat people with psychosis (Santesteban-Echarri et al., 2020). Likewise, psychological treatments such as MCT delivered online, may be an attractive intervention to young people as they are familiar with new technologies, and to women clients due to their caregiving responsibilities (Chiauzzi et al., 2020). It is also promising due to potentially becoming a non-stigmatizing way of receiving psychological treatment and its increased cost-effectiveness (Andersson and Titov, 2014; Osma et al., 2022).

To summarize, a personalized intervention addressed to cover the needs of mothers with psychosis in the care of their adolescent children is necessary. These adolescents have a higher risk of developing a mental health disorder, not just due to genetic reasons but also as a result of their family situation. In addition, adolescence is a crucial stage in the development of metacognitive strategies and the sense of self. In this context, MCT-F has the potential for mothers to work together with their adolescent children on metacognitive strategies, while potentially achieving better functioning and greater understanding to one another, thus enhancing the family relationships.

### 1.5 Study aims

The objective of this study is to evaluate the efficacy of Family Metacognitive Training (MCT-F) in mothers with psychosis and adolescent children in an online group setting to improve family relationships, cognitive insight, symptoms of women with psychosis, and increase their children’s knowledge of the disease. As secondary objectives, we aim to evaluate improvements in metacognition and social cognition, symptoms, protective factors and self-perception of stigma. Finally, we aim to evaluate the feasibility and acceptability of MCT-F in both mothers with psychosis and their adolescent children.

We expect that MCT-F will be effective to improve cognitive insight in mother with psychosis as first hypothesis. As a secondary hypothesis we expect that MCT-F will improvement familiar functioning, metacognition and social cognition variables, as attributional style and Theory of mind, protective factors such as self-esteem, resilience and coping strategies and will decrease self-stigma in mothers. Regarding adolescent children’s participants, we hypothesize that MCT-F will increase children’s knowledge of their mothers’ disease and help them to better understand their mother’s thoughts and their understanding of metacognition and, consequently, to decrease anxiety and depressive symptoms. Furthermore, we expect an improvement in familiar functioning, metacognition and social cognition variables, as well as in protective factors such as: self-esteem, resilience, and coping strategies. More precisely, we expect that all outcomes’ measures will significantly improve from pre- to post-assessment and significant differences when compare with control evaluation. We will control the analysis by cognition functioning, previous trauma and attachment of the mother, presence of the illness before motherhood and attachment of the adolescent with their mother. Finally, we expected that MCT-F Twill be feasible and acceptable to both mothers and their adolescent children.

## 2 Materials and methods

### 2.1 Study design and procedure

The study follows a quasi-experimental design with participants acting as their own control. It was approved by the ethic committees of each participating center and by the Research and Ethics Committee of Parc Sanitari Sant Joan de Déu (PSSJD) (coordinating center) and it conforms to the declaration of Helsinki. All participants meeting the inclusion criteria will receive MCT-F. We will conduct three assessments separated by 11 weeks: an initial no-intervention period of eleven weeks (T1), baseline (T2); and post-intervention (T3).

**Figure 1:**
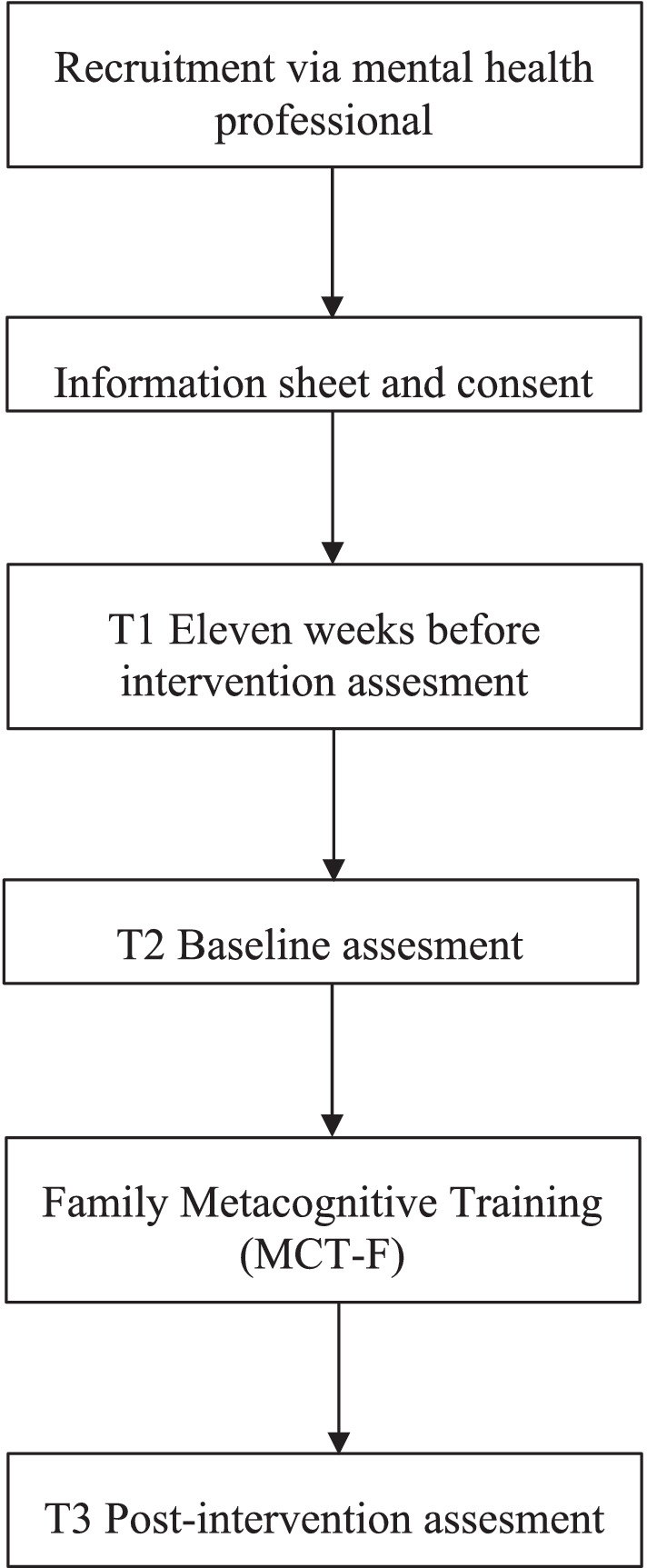
Flowchart of the study.

A complete flowchart of the study can be found in Figure 1. All potentially eligible study participants are recruited from March 2024 to May 2024 by their referring mental health professional from the following 11 participating centers of outpatient mental health services: Hospital de la Santa Creu y Sant Pau (Barcelona), Fundación Jiménez Diaz (Madrid), Corporació Sanitària i Universitària Parc Taulí (Sabadell, Barcelona), Centre d’Higiene Mental de Les Corts (Barcelona), Institut Pere Mata (Reus, Tarragona), Hospital Marqués de Valdecillas (Santander), Parc Salut Mar (Barcelona), Hospital de Mataró (Mataró, Barcelona), Fundació Vidal i Barraquer (Barcelona), Hospital Virgen del Rocío (Sevilla), Mutua de Terrassa (Terrasa, Barcelona), and the coordinating center Parc Sanitari Sant Joan de Déu (Sant Boi, Barcelona). All selected mothers are informed of the study objectives and methodology by their referring mental health professional and sign the required informed consent form. In the case of adolescent participants under the age of 18, in addition to their own consent, the consent of their parents, guardians, or legal representatives is obtained. Then, a psychologist from the research team contacts participating mother by telephone to confirm if she and her adolescent child/children accomplish inclusion criteria. After confirmation of their eligibility, they are enrolled in the study. Specific schedules for the administration of the instruments and the data collection procedure are described below.

### 2.2 Participants

The clinical trial’s sample is comprised of mothers with psychosis with their adolescent children (12–20 years old). Mothers are receiving treatment in one of the outpatient mental health services of the participating groups cited in the previous section.

Inclusion criteria for mother enrolment are as follows: (1) to have a diagnosis of schizophrenia, unspecified psychotic disorder, delusional disorder, schizoaffective disorder, brief psychotic disorder, or schizophreniform disorder (according to DSM-5 criteria); (2) to be a mother to one or more adolescents between 12 and 20 years old; (3) psychopathological stability considering no presence of hospitalization in the previous 3 months.

Exclusion criteria include: (1) to have traumatic brain injury, dementia, or intellectual disability (premorbid IQ ≤ 70); (2) to present PANSS scores ≥5 in hostility, lack of cooperation or suspiciousness, to guarantee a good relationship in the group; (3) to have a substance dependence disorder.

If participant mothers should have more than one adolescent child, all are invited to participate in the study.

Inclusion criteria for adolescents’ enrolment are as follows: (1) to be aged between 12 and 20 years old, (2) to live with their mothers, (3) to be interested in participating in the training with their mothers. The age range of the adolescents in this study was shortened to ensure that they understood and could develop the content of the MCT and also to create age-matched intervention groups of adolescent children.

The exclusion criteria of adolescents include: (1) having a traumatism brain damage or (2) intellectual disability (premorbid IQ ≤ 70).

### 2.3 Measures

Several domains are assessed with mothers and adolescents.

#### 2.3.1 Mothers’ assessment

##### 2.3.1.1 Mothers’ assessment includes the following instruments

All participants complete a sociodemographic questionnaire (age, educational level, marital status, employment status, diagnosis, pharmacological treatment and other data of interest). The duration of untreated psychosis (DUP) is provided by each patient’s referent psychiatrist and relatives. Diagnosis and treatment are collected from their clinical history. Mothers will be asked about the presence of diagnoses in their adolescent children, and it will be taking into account in the analyses as possible confounder variable.

##### 2.3.1.2 Metacognition and social cognition

- The Beck Cognitive Insight Scale (BCIS) (Beck, 2004; Gutiérrez-Zotes et al., 2012) is a self-administered scale composed of 15 items that assesses cognitive insight, yielding a self-reflectiveness subscale and a self-certainty subscale, as well as a Composite Index score. The coefficient of the self-reflectiveness and self-certainty scores was, respectively, 0.59 and 0.62 for the Spanish validation.
- The Cognitive Bias Questionnaire (CBQ) (Peters et al., 2010; Corral et al., 2021) is used for the assessment of the most frequent cognitive biases in psychotic disorders. This questionnaire has a self-administered format, with 30 descriptions of everyday situations, 15 focused on Anomalous Perceptions (AP) and 15 on Threatening Situations (TS). The subject must choose from three options the one that best describes how he or she would think about the situation. Cronbach’s alpha was 0.87 for the total scale (30 items), 0.76 for the AP scale (15 items), and 0.78 (15 items) for the TE scale for the Spanish version.
- Jumping to Conclusions (JTC) reasoning bias (Garety et al., 1991; Dudley et al., 1997). A Spanish version of the Bead Task translated by the research team is used to assess this common cognitive bias in psychosis. Three trials with different conditions are implemented: a probabilistic trial with an 85/15 ratio, a second probabilistic trial with a 60/40 ratio, and salient tasks. JTC bias is considered present if the decision is taken before the third ball.
- The Ambiguous Intentions Hostility Questionnaire (AIHQ) (Combs et al., 2007) is used to evaluate hostile social-cognitive biases in paranoia. It comprises 15 vignettes of negative interpersonal situations and the participants are asked to answer questions about how they would react in each situation. The AIHQ has demonstrated good levels of internal consistency (alpha = 0.84–0.86). The AIHQ has been recently validated for use in the Spanish context and is currently in the process of publishing.
- The Hinting Task (Corcoran et al., 1995; Gil et al., 2012) measures theory of mind. Participants are given three situation in which a character hints to another and they must guess what the character really means in each situation. This task has demonstrated its efficacy as a reliable instrument for discriminating between patients with schizophrenia and controls and has showed a Cronbach’s alpha of 0.69 in the Spanish validation.
- The Faces Test (Baron-Cohen et al., 1997; Huerta-Ramos et al., 2021) measures facial emotion recognition. A total of 20 pictures of a woman displaying an emotion are shown to the participants. Subjects must choose between two options of which emotion better describes each face shown. Psychometric properties of the Spanish version show a Cronbach’s alpha of 0.75.
- The Situational Feature Recognition Test 2 (SFRT-2) (Corrigan et al., 1996; Gómez-Gastiasoro et al., 2018) assesses social perception in patients with schizophrenia. This assessment tool consists of nine situations (five familiar and four unfamiliar) with 14 options for related or non-related actions and goals (six correct responses and eight distractor items for each feature). The Spanish adaptation of SFRT-2 has demonstrated Cronbach’s alphas ranging from 0.83 to 0.90.

##### 2.3.1.3 Clinical symptoms

-The Positive and Negative Syndrome Scale (PANSS) (Kay et al., 1987; Peralta Martín and Cuesta Zorita, 1994) is a structured interview widely used in clinical practice that allows clinical and general symptoms. The PANSS is composed of three subscales: positive (7 items), negative (7 items), and general (16 items), where higher scores indicate higher levels of severity of the symptoms. The PANSS is the most used scale for the assessment of psychotic symptoms. The Cronbach’s alphas for the subscales ranges between 0.72 and 0.80 in the Spanish version.
-The Calgary Depression Scale for Schizophrenia (CDSS) (Addington et al., 1990; Sarró, 2004) is a nine-item structured interview scale that assesses affective symptoms in psychosis. Psychometric properties of the Spanish version show a Cronbach’s alpha of 0.83.
-The Scale of Unawareness of Mental Disorder (SUMD) (Amador et al., 1993; Ruiz et al., 2008) is used to assess awareness of their illness in people with schizophrenia, according to the assessing professional. The scale consists of three general items that evaluates awareness of mental disorder, awareness of the effects of medication and awareness of the social consequences of the disorder; and of 17 items related to specific symptoms, which make up two subscales: awareness and attribution. Good to excellent interrater reliability between evaluators has been reported for the Spanish version, with interclass correlations between 0.82 and 0.91.

##### 2.3.1.4 Social and familiar functioning

- The Family Environmental Scale (FES) (Moos et al., 1974; Fernández-Ballesteros and Sierra, 1989). This questionnaire assesses interpersonal relationships between family members. For this study we use the dimension of family interpersonal relations, composed of 30 items with a true or false response format that measures three dimensions: cohesion (e.g., “In my family, there is a strong feeling of union”), expressiveness (e.g., “At home, we talk openly about whatever we want to discuss”) and conflict (e.g., “Our family members are in conflict with each other”). The Cronbach’s alphas for each dimension are 0.85, 0.80, and 0.86, respectively.-The Satisfaction With Life Scale (SWLS) (Diener et al., 1985; Pons et al., 2000; Muñoz de Arenillas et al., 2010) is a 5-item instrument that assesses the respondent’s degree of satisfaction with their life. The Cronbach’s alpha obtained in the Spanish version is of 0.83.
- The Global Assessment of Functioning (GAF) (Endicott, 1976). This scale measures patients’ general functioning. Clinical and social functioning measures will be included. The validated Spanish version of the DSM-IV was used (American Psychiatric Association, 1996).

##### 2.3.1.5 Protective factors

- The Rosenberg Self-Esteem Scale (RSES) (Martín-Albo et al., 2007) explores through 10 items personal self-esteem understood as feelings of personal worth and self-respect. Cronbach’s alpha for the Spanish version was 0.85.
- The Connor-Davidson Resilience Scale (CD-RISC 10) (Connor and Davidson, 2003; Notario-Pacheco et al., 2011). The 10-item scales assess resilience through three dimensions: self-efficacy-tenacity, personal control and social competence. Cronbach’s alfa for the Spanish version was 0.81.
- The Coping Strategies Inventory (CSI-SF) (Tobin et al., 1989; Cano-García et al., 2007; Tous-Pallarés et al., 2022) is a 16-item questionnaire aimed at measuring the frequency of use of primary coping strategies and the perception of coping self-efficacy. The Spanish version of the CSI-SF has demonstrated good internal consistency, as indicated by a Cronbach’s alpha of 0.86.
- The Self Stigma Questionnaire (SSQ) (Ochoa et al., 2015) assesses self-stigma through 14 items and it has been validated in people with psychosis. The results for the Spanish version indicated good psychometric properties, with Cronbach’s alpha ranging between 0.75 and 0.90.

##### 2.3.1.6 Cognitive functioning

- The Vocabulary subscale from Wechsler Adults Intelligence Scale (WAIS) (Wechsler, 2008) is used to explore premorbid intelligence quotient (IQ) in FEP (González-Blanch et al., 2011). It is a task based on an individual’s general knowledge of linguistic information (phonology and semantics), which has been generally associated with crystallized intelligence.
- The Trail Making Test (TMTA-B) (Reitan, 1958; Sánchez-Cubillo et al., 2009) is a test integrating two parts: part A measures the speed of processing and part B measures mental flexibility. In each test the participant is asked to draw a line between 24 consecutive circles that are randomly arranged on a page. The TMT-A uses all numbers, whereas the TMT-B alternates numbers and letters, requiring the patient to switch between numbers and letters in consecutive order. The score represents the amount of time required to complete the task. The average for the TMT-B is 75 s, with deficiencies noted >273 s for the Spanish version.
- The Screen for Cognitive Impairment in Psychiatry (SCIP) (Purdon, 2005; Pino et al., 2008) assesses cognitive difficulties in patients with severe psychiatric disorders. Spanish version showed an average time for administration of 16.02 (SD = 5.01) and an internal consistency Cronbach’s alpha value of 0.73.

##### 2.3.1.7 Attachment and trauma

-The Psychosis Attachment Measure (PAM) (Berry et al., 2006; Sheinbaum et al., 2013) is a 16-item self-report scale designed to measure adult attachment in people with psychosis. For the Spanish version of the PAM, Cronbach’s alpha coefficients of 0.81 and 0.78 were found for the anxiety and avoidance subscales, respectively.
-The Maltreatment and Abuse Chronology of Exposure Scale (MACE) (Teicher and Parigger, 2015) consists of 52 items that measures severity of exposure to ten types of maltreatment and provides an overall severity score and multiplicity score. Prior to this study, the MACE was adapted and translated into Spanish has been utilized in previous studies (Montoro et al., 2023).

#### 2.3.2 Adolescents’ assessment

Adolescent children assessment includes the following instruments:

All adolescents complete a sociodemographic questionnaire.

##### 2.3.2.1 Metacognitive and social cognition

Adolescents are assessed with the same battery of instruments as their mothers.

##### 2.3.2.2 Clinical symptoms

-The Strengths and Difficulties Questionnaire (SDQ) (Goodman, 1997; Fonseca-Pedrero et al., 2011). The 25-item scale provides scores for 5 subscales including emotional symptoms, conduct problems, symptoms of hyperactivity/ inattention, peer problems, and prosocial behavior. In the Spanish version, Cronbach’s alpha ranged from 0.58 (behavioral problems) to 0.71 (emotional symptoms).

##### 2.3.2.3 Social and familiar functioning

Adolescents are assessed with the with the FES as their mothers because it has been validated for the age population.

Satisfaction with Life Scale for Children and Adolecents (SWLS-N) (Diener et al., 1985; Sandín et al., 2015) is an age-downward version of the measure of life satisfaction developed by Diener. It is a 5-item, self-report instrument in which respondents are asked to indicate the degree to which t are satisfied each statement is true of their satisfaction with their life. The Spanish version of SWLS scale has demonstrated an alpha de Cronbach of 0.89 (García-Escalera et al., 2017).

The Children’s Global Assessment Scale (CGAS) (Shaffer, 1983) is an adaptation of the Global Assessment Scale (GAS) that assesses the level of functioning for a child or adolescent during a specified time period. It consists of a single score that ranges from 1 (most impaired) to 100 (healthiest). The CGAS has obtained good levels of reliability, with diagnostic agreement values between judges (kappa coefficient) higher than 0.90 (Santamarina-Perez et al., 2020).

##### 2.3.2.4 Protective factors

Adolescents are assessed with the same battery of instruments as their mothers. These instruments have been validated for the age population, except for the Self-Stigma Questionnaire (SSQ), which is not administered.

##### 2.3.2.5 Attachment

- The short version of Cartes, Modèles Indivuels de Relation (CaMir-R) (Balluerka et al., 2011) is a questionnaire aimed at measuring attachment cognitions. It is based on the subjects’ evaluations of past and present attachment experiences and family functioning. The Spanish version of the CaMir-R has demonstrated good internal consistency, as indicated by a Cronbach’s alpha of 0.81.

##### 2.3.2.6 Cognitive functioning

- The Vocabulary subscale from Wechsler Adults Intelligence Scale (WAIS) (Wechsler, 2008) is used to explore the premorbid intelligence quotient (IQ) in adolescents aged 16 years or older. For those participants younger than 16, the Vocabulary subscale of the Wechsler Intelligence Scale for Children (Wechsler, 2014) is used.
- The Matrix Reasoning Task of the Wechsler Intelligence Scale for Children (Wechsler, 2008, 2014) measures visual processing and abstract, spatial perception.
- The Trail Making Test (TMTA-B) (Reitan, 1958; Sánchez-Cubillo et al., 2009) is applied using scales from the age range of the study.

##### 2.3.2.7 Feasibility and acceptability

All mothers and adolescent’s participants are also asked about the perceived value and usefulness of the intervention and the delivery format through a brief self-reported questionnaire divided into two parts. The first part consists of 8 generic items on an 11-point Likert scale from 0 (“not at all”) to 10 (“completely”). The second part consists of 8 short open-ended questions. Finally, we will evaluate the impact of the program’s aim to increase adolescent children’s knowledge of their mothers’ disease with the following items: “To what extent do you feel you understand your mom’s disease before participating in the training?” and “To what extent do you feel you understand your mom’s disease after participating in the training?” with Likert-type response options (from 0 “Not at all” to 5 “Completely”).

In addition, after each session participants are asked to answer two questions to measure the perceived usefulness and enjoyment of each module: “How much did you enjoy it?” and “How useful did you find it?”

### 2.4 Data collection

Assessments are conducted at the three afore mentioned time points. Evaluators have been trained in psychological evaluation. The neuropsychological assessment will be carried out in the presence of the participants. The rest of the assessment will be conducted on another day and online to increase the feasibility and prevent fatigue due to the large number of assessment instruments. The assessment of the mother and the adolescent will be conducted separately but the same evaluator. Instruments used in each evaluation and the corresponding informant (mother or adolescent) is reported in Table 1. Types and dosages of the mothers’ medication as well as attendance to psychological and/or pharmacological treatment for the adolescent children will be recorded at the three time points of assessment.

**Table 1:** Measures used in the study with mothers and their adolescent children.

Instruments	Informants		Time of assessment		
	Mothers	Adolescent	T1	T2	T3
Inclusion/exclusion criteria	X	X	X		
Informed consent	X	X	X		
Sociodemographic and clinical questionnaire	X	X	X		
Clinical symptoms			X	X	X
PANSS	X		X	X	X
CDSS	X		X	X	X
SUMD	X		X	X	X
SDQ		X	X	X	X
Metacognition and social cognition					
BCIS	X	X	X	X	X
CBQ	X	X	X	X	X
JTC	X	X	X	X	X
AIHQ	X	X	X	X	X
The Hinting Task	X	X	X	X	X
Faces Test	X	X	X	X	X
SFRT-2	X	X	X	X	X
Familiar and social functioning					
FES	X		X	X	X
GAF	X	X	X	X	X
SWLS	X		X	X	X
CGAS		X	X	X	X
SWLS-N		X	X	X	X
Protective factors					
RSES	X	X	X	X	X
CD-RISC 10	X	X	X	X	X
CSI-SF	X	X	X	X	X
SSQ	X		X	X	X
Attachment and trauma					
PAM	X		X		
MACE	X		X		
CAMI-R		X	X		
Cognitive functioning					
SCIP	X		X		X
TMT (A)	X	X	X		X
TMT (B)	X	X	X		X
WAIS IV	X	X	X		
WISC		X	X		
Matrix		X	X		X
Feasibility and acceptability^a^	X	X			X

AIHQ, The Ambiguous Intentions Hostility Questionnaire; BCIS, Beck Cognitive Insight Scale; CAMI-R, The short version of Cartes, Modèles Indivuels de Relation; CBQ, Cognitive Bias Questionnaire; CD-RISC 10, The Connor-Davidson Resilience Scale; CDSS, The Calgary Depression Scale for Schizophrenia; CGAS, Child Global Assessment Scale; CSI-SF, The Coping Strategies Inventory; CTQ-SF, Childhood Trauma Questionnaire; FES, Family Environmental Scale; GAF, The Global Assessment of Functioning; JTC, Jumping to Conclusions reasoning bias; MACE, Maltreatment and Abuse Chronology of Exposure; PAM, Psychosis Attachment Measure; PANSS, Positive and Negative Syndrome Scale; RSES, Rosenberg Self-Esteem Scale; SCIP, The Screen for Cognitive Impairment in Psychiatry; SDQ, The Strengths and Difficulties Questionnaire; SFRT-2, The Situational Feature Recognition Test 2; SSQ, Self-Stigma Questionnaire; SUMD, Scale of Unaweressness of Mental Disorder; SWLS, The Satisfaction With Life Scale; SWLS-N, Satisfaction with Life Scale for Children and Adolecents; T1, An initial no-intervention period of eleven weeks; T2, Baseline; T3, Post-intervention; TMT-A, The Trail Making Test – A; TMT-B, The Trail Making Test – B; WAIS IV, Wechsler Adults Intelligence Scale IV; WISC, Wechsler Intelligence Scale for Children. ^a^Feasibility and Acceptability will be assessed in all sessions of the MCT-F by both participants.

### 2.5 Interventions

The intervention consists of an adaptation of classical MCT to a family setting (MCT-F), that is, for mothers with psychosis and their adolescent children in a group setting with other peers. Metacognitive training for psychosis is a psychological intervention that combines psychoeducation with a cognitive behavioral intervention in a metacognitive approach (Moritz and Woodward, 2007). It consists of a normalizing approach to work on cognitive biases which present in the general population but that are more prevalent in people with psychosis, as they are involved in the appearance and maintenance of delusions (Freeman, 2007; Moritz and Woodward, 2007).

Before the quasi-experimental study, a first stage consisted of adapting MCT to this population using the first six steps of the ADAPT-ITT method through a participatory approach, which included the first-person perspective and involved qualitative and quantitative methods. While the core components of the original MCT intervention were retained, patients, relatives, adolescents, and experts participated to ensure that MCT-F was appropriate and acceptable (Espinosa et al., 2024).

The final version of MCT-F consists of 11 online weekly group sessions lasting 60 min. The groups will be composed of 3–4 mothers with psychosis, their adolescent children and two therapists. This group size is large enough to allow for some participants’ absence from sessions without leaving those attending feeling exposed. It is also manageable for two facilitators. Furthermore, participants will be allocated to their groups based on the teenagers’ ages (aged 12–16 and 16–20) to adapt examples and vocabulary and so they feel more comfortable sharing experiences. Mothers and adolescents will be able to attend sessions from the same room or from separate places, to facilitate attendance and the intimacy of both participating groups. Therapists will also be local community references to the participating mothers to enhance adherence and assure the inclusion of the study’s information in their clinical records. Although there is no minimum number of group sessions that participants must attend, the attendance of each participant will be recorded.

The material available to use during MCT-F is made up of power-point presentations. Each module contains therapeutic material including psychoeducational information, exercises, case examples and demonstrations. The first session is added as a psychoeducation session to explain the psychotic illness, metacognition, and training objectives. Seven of the therapeutic modules address cognitive bias and errors that are frequently seen in problem-solving in psychosis. The other two modules work with depression, self-esteem, and self-stigma. Lastly, we added an additional session, after third session, with mothers and adolescent children attending separately to facilitate speaking openly about feelings or concerns without their relatives.

All mother participants will also receive their treatment as usual (TAU). In order to account for differences in TAU over the different centers the mothers participate in, we will collect the specific treatment characteristics of the TAU offered in each institution.

### 2.6 Sample size calculation

Taking as a reference previous data on effectiveness of MCT in women samples (Salas-Sender et al., 2020), the sample size necessary for the study to have adequate power has been estimated. With an alpha of 0.05, a power of 0.90, a difference between pre-treatment and post-treatment in cognitive insight (self-certainty) of 2 points in the intervention vs. −0.48 in the control, and with a SD of 4.14 in the reference group, as well as considering possible losses of 25%, the final sample calculation is estimated to be of 48 mothers (with their respective children) in total.

### 2.7 Data analysis

The main analysis variable will involve the difference between scores in the control assessment and intervention assessment for the cognitive insight assessed in the mothers with psychosis. Secondary outcomes will be the changes in other assessments of metacognition and social cognition variables, familiar and social functioning, cognitive distortions, cognitive functioning, protective factors, and self-stigma.

The changes in the cognitive insight scales’ scores will be analyzed using repeated measures regression methods. Changes in the scores of symptoms, metacognition and social cognition, social functioning, understanding of the disease, self-esteem, resilience and coping strategies in adolescent children will be analyzed using repeated measures regression methods, with the final score of the scale as the dependent variable. Two additional analyses will be carried out to examine the robustness of the results. First, the intention-to-treat analysis will be performed after replacing missing values for the dropout cases using the method “last observation carried forward” (LOCF). Next, the analyses will be performed only with data from participants who complete the treatment and who have been evaluated at both time points. Finally, the satisfaction of patients undergoing group treatment will also be analyzed.

All the analyses will be controlled by medication changes, cognitive functioning, attachment, trauma, start of the illness before motherhood and type of TAU (in mothers), and the presence of mental disorders or attendance to psychological and/or pharmacological treatment (in adolescents).

## 3 Discussion

To our knowledge, this is the first study aiming to adapt and assess the effectiveness of Family Metacognitive Training (MCT-F) in a sample of mothers with psychosis and their adolescent children. We will also evaluate the feasibility and acceptability of the adapted intervention. It is novel in this context, in that not only does it address patient symptoms and cognitive awareness, but also the family relationship. It also aims to improve the children’s understanding of the disease and their functioning. As secondary objectives, MCT-F is seeking to increase metacognition and social cognition, improve symptoms, protective factors, and self-perception of stigma in both family members.

Considering the efficacy of MCT in women with first-episode psychosis (Ochoa et al., 2017), we similarly expect mothers with psychosis who receive MCT-F will improve in cognitive insight, as well as symptoms and other metacognitive and social cognition measures. In addition, women with FEP have shown better responses to psychological interventions, not only in terms of improvements but in compliance and motivation, so we expect the results in these mothers to be even better (Villeneuve et al., 2010). Additionally, we hypothesize that mothers that receive MCT-F will increase their familial and social functioning, and protective factors such as self-esteem, resilience and coping strategies, and will see reductions in self-stigma. MCT (Moritz and Woodward, 2007) focuses on different cognitive biases, social cognition variables (ToM, emotional perception, and attribution bias), depressed mood and low self-esteem in paranoid ideation. All these aspects can help mothers by offering them alternative ways of interpreting situations and experiences, while reducing social cognition difficulties by improving their ability to understand their children’s mental states.

Although no previous research has been done in the lines of the present study, other interventions involving children of parents with other mental disorders found that psychoeducation improves depressive symptoms, familiar functioning, understanding of the parent’s disorder, resilience and coping strategies (Tapias et al., 2021). We hypothesize that this intervention could be equally helpful to the adolescents, to better understand their mother’s thoughts and their understanding of metacognition and, consequently, to decrease anxiety and depressive symptoms. Furthermore, we expect an increase in familiar and social functioning, as well as in protective factors such as self-esteem, resilience and coping strategies. While there is a gap in study of metacognition and social cognition in the adolescent population (Tapias et al., 2021), we have found evidence for different social cognitive and metacognitive affectations as a function of having a mother or a father with a history of mental illness (Mendoza-García et al., 2022). Thus, we consider high-risk adolescents may benefit from the group MCT-F and improve in these areas.

Some features of the present study can be considered assets. First of all, it contributes to the still scarce literature on the efficacy of psychological intervention for mothers with psychosis and their adolescent children. A second strength of our study is the participatory nature of the adaptation process which involved patients, relatives, adolescents, mental health professionals and researchers. Using a variety of methods (e.g., consensus group discussions, interviews, desk reviews), we triangulated data to inform evidence-based adaptations. Thirdly, contrary to most similar studies aiming to evaluate the efficacy of these interventions (Radley et al., 2022c), this study will assess feasibility and acceptability not only in mothers, but also in the adolescent children. Furthermore, from our point of view, evaluating the impact of familiar interventions on metacognition and social cognition, in addition to traditional clinical variables, cognition functioning, protective factors and self-stigma perception is also especially important to consolidate this approach as an effective therapy. Finally, MCT-F has the advantages of an online-based treatment, including better cost-effectiveness and addressing current barriers to access mental health services in a non-stigmatizing way. Further, it adds the benefit of encouraging the recruitment of patients residing in remote areas without specialized care resources.

Once MCT-F’s clinical utility is confirmed, these findings will have important clinical implications. First, the availability of psychological therapy would help these families to deal with these cognitive distortions that are more present in the disease and that have therefore the exacerbation of symptoms and problems in social and family relationships (Radley et al., 2022a). Thus, an intervention such as MCT-F targeting symptoms, social cognition, and metacognition difficulties could improve mothers’ awareness of their children’s mental states and difficulty in providing adequate care to them and, consequently, prevent loss of custody. On the other hand, working on the presence of these cognitive distortions in a normalized way in those high-risk adolescents can be useful as a preventive approach to avoid the appearance of these distortions and consequently the symptoms associated with schizophrenia (Riches et al., 2019; Seeman, 2021).

Study limitations must also be acknowledged. The main limitations of the present study are the lack of a comparison group and the small sample size. A quasi-experimental design was chosen given that impairments in recruiting and retaining people with psychosis and their families are well-established in previous research, potentially biasing clinical research samples (Polillo et al., 2021). Additionally, the comprehensive assessment included, encompassing numerous scales and questionnaires, may impact the quality of participant responses and affect the results. To address this, the assessment will be conducted in different sessions, featuring varied questions and assessments to sustain participant engagement. Another limitation of the study is the potential difficulty isolating the impact of MCT-F on outcome measures. We expect that participants acting as their own control and the statistical analyses mentioned above can increase the robustness of the study results and address these limitations. In addition, the online design nature of this trial may encourage adherence, as it allows a better adjustment to adolescents’ school schedules and the use of new technologies is well accepted by them. To confirm this, we will collect the participants’ rate of attendance to control for this variable. However, the use of online modality could also reinforce family secrecy or feelings of isolation. Also, the absence of inclusion of other family members (e.g., the other caregiver) may hinder changes in the environment. In this sense, this pilot study would be the first step toward obtaining preliminary data.

If the results of this study show that MCT-F is effective, the next steps will include larger sample sizes, follow-up periods, comparison of face-to-face and online modalities, exploration of active or passive control conditions to be able to clearly attribute effects to MCT-F, and potentially adding more peer groups sessions (where mothers and adolescent children attend separately) or other family member (such as co-parent). Finally, as there appears to be a growing tendency for men with psychosis to become fathers, we also consider that fathers with psychosis could especially benefit from MCT-F which can be explored in future trials.

## 4 Conclusion

To summarize, a personalized intervention addressed to meet the needs of women in care of their adolescent children is necessary. Currently no therapy along these lines has been designed or evaluated worldwide. Should Family Metacognitive Training prove effective for mothers with psychosis and their adolescent children, it could contribute to expanding the psychotherapeutic approaches available for these families. MCT-F could both help to understand one another and to deal with daily problems for both family members, while also improving symptoms in mothers and decrease their children’s higher risk of developing mental disorders.

## Ethics statement

This study has been evaluated and approved by each of the Ethics Committees of the participating centers. This clinical trial has been registered (clinicaltrials.gov) (identifier: NCT05358457). All participants are being provided with an information sheet explaining the objectives and procedure of the study as well as the confidentiality of the data collected. All participants will be asked in writing for their consent in accordance with the Declaration of Helsinki and Law 14/2007 on Biomedical Research.

## Author contributions

SO: Writing – original draft, Writing – review & editing, Funding acquisition, Investigation, Project administration, Supervision. VE: Writing – original draft, Writing – review & editing, Methodology. RL-C: Writing – review & editing, Conceptualization, Methodology. IM: Writing – original draft. AHB: Writing – original draft. IB: Writing – review & editing. AB: Writing – review & editing. TP: Writing – review & editing. LD-C: Writing – review & editing. MC: Writing – review & editing. AG-R: Writing – review & editing. MV-R: Writing – review & editing. AG-Z: Writing – review & editing. CP-S: Writing – review & editing. CrM: Writing – review & editing. JG: Writing – review & editing. BP: Writing – review & editing. FC: Writing – review & editing. MR: Writing – review & editing. EN: Writing – review & editing. PV: Writing – review & editing. MS-S: Writing – review & editing. AA: Writing – review & editing. RA-A: Writing – review & editing. EP: Writing – review & editing. MC-R: Writing – review & editing. NG-T: Writing – review & editing. ClM: Writing – review & editing. LM-L: Writing – review & editing. JC: Writing – review & editing.

## Footnotes

^1^ www.uke.de/mct
